# Epithelial Plasticity During Human Breast Morphogenesis and Cancer Progression

**DOI:** 10.1007/s10911-016-9366-3

**Published:** 2016-11-04

**Authors:** Saevar Ingthorsson, Eirikur Briem, Jon Thor Bergthorsson, Thorarinn Gudjonsson

**Affiliations:** 1 Stem Cell Research Unit, Biomedical Center, School of Health Sciences, University of Iceland, Reykjavík, Iceland; 2 Department of Laboratory Hematology, Landspitali, University Hospital, Reykjavík, Iceland

**Keywords:** 3D cell culture, Breast cancer, EMT, Stem cells, Plasticity

## Abstract

Understanding the complex events leading to formation of an epithelial-based organ such as the breast requires a detailed insight into the crosstalk between epithelial and stromal compartments. These interactions occur both through heterotypic cellular interactions and between cells and matrix components. While in vivo models may partially capture these complex interactions, there is a need for in- vitro models to study these events. In this review we discuss cell-cell interactions in breast development focusing on the stem cell niche and branching morphogenesis. Given the recent understanding that the basic developmental events underlying branching morphogenesis are closely related to pathways important to cancer progression, i.e. epithelial plasticity and epithelial to mesenchymal transition (EMT), we will also discuss aspects relevant to cancer progression. In cancer, the adoption of mesenchymal phenotype by the malignant cells allows stromal invasion and subsequent intravasation to blood- or lymphatic vessels, a route that is a prerequisite for metastasis. A number of publications have demonstrated that tumor initiating cells, sometimes referred to as cancer stem cells adapt an EMT phenotype that renders them more resistant to apoptosis and drug therapy. The mechanism behind this phenomenon is currently unknown but this may partially explain relapse in breast cancer patients. Increased understanding of branching morphogenesis in the breast gland and the regulation of EMT and its reverse process mesenchymal to epithelial transition (MET) may hold the keys for future development of methods/drugs that neutralize the invading properties of cancer cells.

## Introduction

Epithelial cells serve multiple functions in the human body. These include barrier functions (skin, trachea), hormonal secretion (pituitary gland, adrenal glands and Langerhans islands in the pancreas), exocrine secretion (prostate, pancreas, salivary gland, breast gland), absorption, filtration and gas exchange (intestine, kidneys and lungs). To serve its function, epithelial cells have adhesion properties that generate tight layer(s) of squamous, cuboidal or columnar epithelium dependent on location and function within the human body. Due to the immediate exposure of epithelial tissue to external environment cellular remodeling and renewal occurs relatively fast, meaning that new cells are continuously replacing older cells. Epithelial organs therefore contain stem cells that are responsible for the continuous cellular remodeling [1]. Furthermore, it has been suggested that epithelial cancers originate in these stem cells or cells that have acquired stem cell properties [1–3]. The female breast gland is a unique organ in that most of its development occurs postnatally. The breast gland undergoes repeated cycles of cell proliferation, differentiation and involution from menarche to menopause at which point hormonal signals, or lack thereof, cause cell death by triggering a combination of apoptosis and senescence [4–6]. These cellular remodeling processes are most prominent during pregnancy and lactation when the breast gland becomes fully differentiated.

The branching nature of the epithelial ducts in the breast requires a level of phenotypic plasticity enabling cells to invade the underlying stroma. Cells need to transit from robust epithelial cell-cell binding to a more mobile state to facilitate migration. The cells can achieve this using distinct mechanisms including collective migration [7, 8] or epithelial to mesenchymal transition (EMT) where leading cells at the tip of the branching structures acquire mesenchymal characteristics that facilitate migration into the surrounding stroma [9]. EMT is a fundamental process in normal embryonic development, particularly during formation of mesoderm, neural crest formation and heart valve development [10]. EMT is also an important process during wound healing. Finally, EMT has been closely linked with breast cancer progression where tumors of certain sub-groups have been demonstrated to be driven by cancer stem cells that have acquired mesenchymal traits that greatly enhances their tumorigenicity and metastatic potential [9, 11, 12]. In this review we will focus on the cellular and molecular mechanisms of breast morphogenesis and EMT and its reversed process mesenchymal to epithelial transition (MET) and how these processes can be recapitulated in stromal-rich three-dimensional cell culture assays. In addition, we will discuss the clinical relevance of EMT, MET and cancer stem cells in breast cancer in terms of diagnostic value, prognosis and therapeutic application.

## Normal Mammary Gland Development

**Fig. 1:**
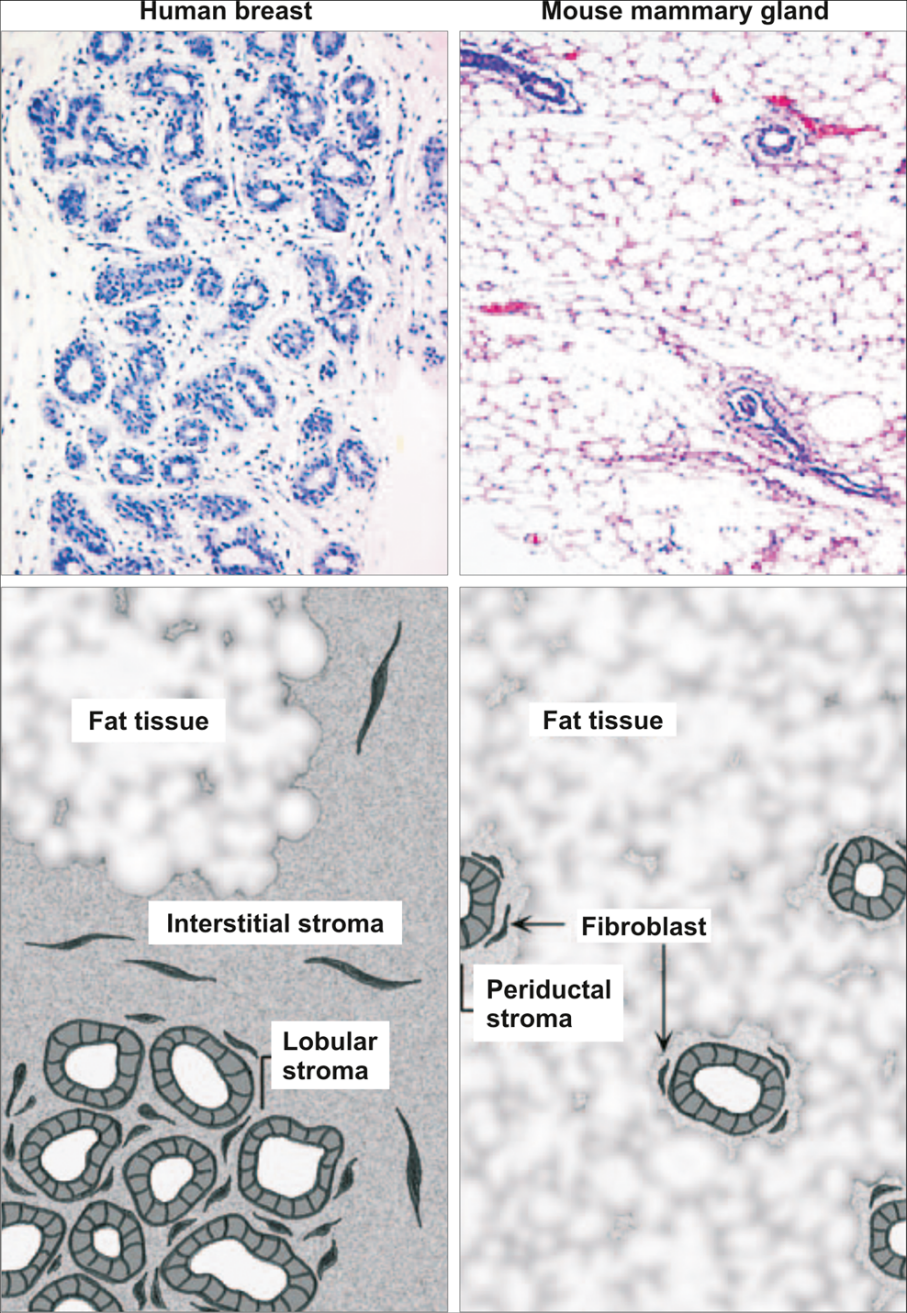
Histological differences between the human breast and mouse mammary gland. The functional unit of the human breast gland is the terminal duct lobular unit (TDLU). This structure is composed of multiple acini and small ductules and is embedded in a collagenous stroma. The stroma can be divided in two, the interstitial stroma, between TDLUs and ducts, and the looser cellular-rich lobular stroma, within TDLUs. The Human mammary tissue along with the stroma, is in turn embedded in fat tissue. In mice, the mammary gland is composed of a series of branching ducts, terminating in the terminal end bud (TEB). Compared to human breast tissue, the collagenous stroma surrounding the mouse mammary epithelium has a much smaller volume (periductal stroma), so the epithelial structure is enclosed by fat tissue. Adapted from Parmar et al. [13] with permission

The breast gland, somewhat uniquely, develops in different stages separated in time often by years or decades. In early embryonic mammary gland development, the formation of the mammary epithelial placodes in the skin is a critical event. These epithelial buds invade the underlying mesenchyme to form a rudimentary ductal systems embedded in stroma that develops along with the mammary epithelium [13]. Most studies focusing on mammary development are based on mouse models due to ease of access and great availability of differently mutated mouse strains. Although critical developmental events may be conserved through evolution there are notable differences between humans and mice. Importantly, there are differences regarding the stroma surrounding the branching epithelium. In humans, the epithelium is embedded in cell-rich collagenous stroma while in mice the stromal component is mostly adipose tissue commonly referred to as the mammary fat pad [13, 14] (Fig. 1). In addition, while the structure of the glandular tissue is fundamentally similar in mice and humans, the human branching tree displays a markedly higher complexity that is partly explained by its greater size. The terminal mammary epithelial structures are different in mice and humans. In mice the epithelial ducts terminate in the end bud (TEB). In contrast the human female breast is more elaborate; terminating in ductal lobular units (TDLUs) that are characterized by clusters of acini and small ductules [14].

In humans, breast glands are similar in both sexes until the onset of puberty. In women, the second phase of development occurs during puberty. This phase is characterized by marked stromal and epithelial proliferation via hormonal stimulation. Estrogen signaling through the estrogen receptor α (ERα) is the primary force driving ductal branching and elongation, while progesterone signaling through its cognate receptor is required for proliferation and terminal differentiation in the TDLU [15]. The epithelial tubes elongate extensively at this stage and undergo more pronounced branching than during the embryonic stage.

The final major developmental phase involves pregnancy and lactation. During pregnancy, further branching occurs and the cells within the TDLUs proliferate extensively to prepare for the final functional stage of lactation. The luminal epithelial cells finally reach their terminally differentiated state during the lactation period. At all other time periods, the luminal epithelial cells are considered to be in an immature, non-functional state. After lactation, the gland undergoes involution, characterized by massive epithelial apoptosis and return to a near pre-pregnancy non-functioning state. It is well established that the duration of the interval between the pubertal stage and first pregnancy is major risk factor for breast cancer. In that regard it has been proposed that repeated hormone stimulation of the stem cell niche in the course of repeated menstrual cycles prior to first pregnancy may be an important early event in breast carcinogenesis [16]. Overall, pregnancy leads to a long-term protective effect against breast cancer, presumably via a depletion of the stem cell reservoir [17].

The putative mammary epithelial stem cell is responsible for growth, maintenance and late developmental generation and re-generation (at repeated pregnancies) seen through these distinct different phases of early and late development. The mouse mammary stem cell has been fairly well characterized using genetic techniques for tracking and transplantation assays in cleared mammary fat pad model [18, 19]. This cell type, characterized by a Lin^−^CD29^hi^CD24^+^ immunophenotype, can at a single cell level regenerate the mouse mammary epithelium. The immature mammary gland stem cells have been located in the so-called peripheral cap cells of the mouse terminal end buds. Furthermore, in addition to the mammary stem cell, there is evidence for a hierarchical structure within the mammary epithelial tree, with both lobule- and duct-restricted progenitor cells [20]. While progenitor cells exist within the luminal population, these cells and their descendants have a relatively short lifetime in the gland, and are replaced in the course of a few months. The long term repopulating cells within ducts reside in the myoepithelial/basal layer. These cells give rise to transit amplifying cells within both luminal and myoepithelial layers as evidenced using lineage tracing, clonal expansion of cells that express a single reporter fluorochrome [21].

**Fig. 2:**
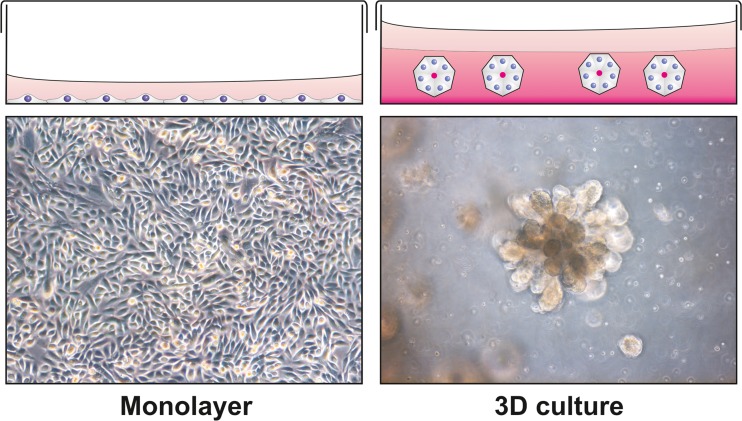
Breast morphogenesis in 3D cell culture. During conventional cell culture, cells proliferate in a monolayer forming a sheet of cells at the bottom of the culture dish. This is an environment that is highly abstract to cells that in vivo find them self in a 3D environment. This may have great effects on gene expression resulting in altered phenotype. When seeded in a 3D matrix such as reconstituted basement membrane (rBM) matrix the same cells can undergo drastically different morphogenesis, forming polarized spheres with a central lumen, or as for certain cell lines, branching morphogenesis

In comparison to the mouse, the human mammary epithelial stem cell is less well defined. There are, however, several studies that have isolated and/or characterized human breast epithelial cells with stem cell properties that have to a varying degree been able to recapitulate the human breast epithelial phenotype [22–25]. Using cells from reduction mammoplasties cultured in three dimensional (3D) reconstituted basement membrane (rBM) matrix we and others have been able to isolate and characterize putative human mammary stem cells that are able to regenerate a branching bilayered epithelium (Fig. 2) with lineage committed luminal and myoepithelial cells [23, 26]. These cells have a subrabasal location in the breast gland, residing between the myoepithelial and luminal cells. They were initially isolated on the basis of epithelial specific antigen (ESA/EpCAM)) positive and sialomucin (MUC-1) negative immunophenotype. Understanding how the breast epithelium including breast epithelial stem cells interacts with the surrounding stroma is crucial as these studies may shed light on breast morphogenesis, cellular remodeling and heterotypic interactions during breast cancer progression.

## Epithelial-Stromal Interactions

The growth and branching morphogenesis of breast epithelium is under instructive influences of the surrounding stroma. The breast stroma is composed of collagenous extracellular matrix (ECM) and resident stromal cells including fibroblasts, immune cells, adipocytes and endothelial cells [14, 27]. In contrast, the mouse mammary gland is enclosed by fat tissue with fewer stromal cells in direct contact with epithelial tissue [13, 14] (Fig. 1). Fibroblasts and immune cells are acknowledged as important players in breast morphogenesis and neoplasia [28–30] while endothelial cells have been largely neglected except in angiogenesis during cancer growth. Fibroblasts are the main producers of ECM and they are also known to secrete growth factors and remodeling proteins such as matrix-metalloproteases (MMPs) that stimulate proliferation and branching morphogenesis of breast epithelium [31]. It has also been shown that priming of mouse tissue with human fibroblasts before implantation of human epithelial xenografts improves the mouse model for studying molecular signals during human breast branching morphogenesis [32].

Endothelial cells have previously been pictured as passive constituents of the conductive system transporting oxygen and nutrients towards tissues and waste products away. In addition, they have more recently been identified as critical regulators of the stem cell niche and organogenesis in many tissues, such as brain, liver and pancreas [33–35]. In the prostate, post-castration stimulation with androgens expands the vasculature before epithelial recovery suggesting that endothelial-derived signals may be vital for epithelial growth [36]. We have recently shown that endothelial cells facilitate growth and branching morphogenesis of prostate epithelial cells when cocultured in reconstituted basement membrane (rBM) [37]. Evidence supporting the role of the endothelium in modulating the branching epithelial morphogenesis in the lung has also emerged. Like in the prostate, we have shown that endothelial cells profoundly stimulate growth and branching morphogenesis of lung epithelium in 3D culture. In this assay a basal-like human pulmonary epithelial cell line (VA10) with stem cell properties [38] undergoes branching morphogenesis when cocultured with endothelial cells. These branching colonies were reminiscent of tubulo-alveolar-like structures [39]. Recently, a critical endothelial- epithelial signaling interaction was reported in a study using a mouse lung model. Recovery of lung tissue following pneumonectomy was shown to be mediated by an endothelial-to-epithelial signaling cascade involving the matrix as an intermediary player. The pulmonary endothelial cells were shown to express and secrete matrix metalloproteinase 14 (MMP14) in response to vascular endothelial growth factor (VEGF) and fibroblast growth factor (FGF) stimulation. The endothelial derived MMP14 contributed by generating EGF-like ligands by cleaving native laminin 5-γ2 in the matrix thus activating EGF receptors (EGFR) expressed on the surface of alveolar epithelial cells [40].

Analogous to the lung, we have shown that microvessels in a normal human breast are abundant and in close proximity to the epithelial cells of the TDLUs [41]. Studies have confirmed that during the menstrual cycle and pregnancy, vascularity of the normal mammary fluctuates in correlation with hormonal levels [42]. Interaction between human umbilical vein endothelial cells (HUVEC) and premalignant breast epithelial cells has been shown to support proliferation of endothelial cells and induce ductal-alveolar branching morphogenesis and hyperplasia of premalignant breast epithelial cells [43, 44]. We have improved the isolation protocol and conditions for long term culture of breast endothelial cells (BRENCs) [41]. Using BRENCs we have developed a novel three dimensional co-culture model, where primary breast endothelial cells are seeded with epithelial cells in 3D rBM. Using this assay we have shown that BRENCs stimulate proliferation of both primary luminal- and myoepithelial cells. Furthermore, the endothelial cells stimulated the growth and cloning efficiency of normal and malignant breast epithelial cell lines and this was contributed by soluble factors [45].

**Fig. 3:**
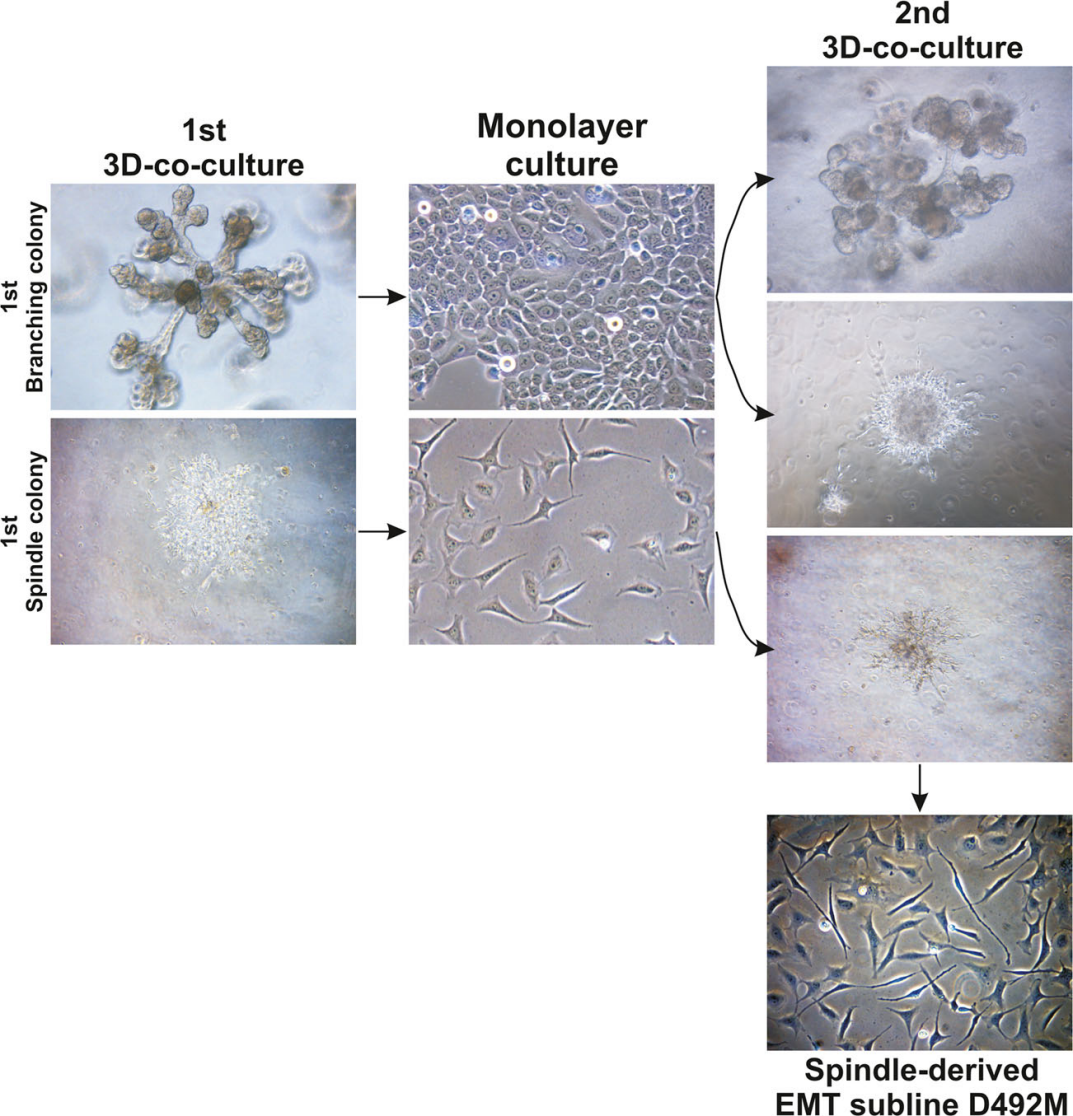
Generation of the D492M cell line. The epithelial cell line D492 was established from normal breast tissue [23]. When cultured in 3D, it forms branching colonies reminiscent of the TDLU. In co-culture with breast endothelial cells, this cell line can form mesenchymal colonies. When branching colonies are isolated from 3D culture and re-plated, they form both branching and mesenchymal colonies during secondary co-culture, while the mesenchymal colonies only give rise to additional mesenchymal colonies. A mesenchymal cell line (D492M) was established from a mesenchymal colony using single cell cloning. Adapted from Sigurdsson et al. [46] with permission

When BRENCs are co-cultured in 3D rBM with D492, a breast epithelial cell line with stem cell properties, we observe increased branching morphogenesis, supporting the data that BRENCs contribute to regulation of branching epithelial morphogenesis. D492 was initially established from EpCAM positive, MUC1 negative suprabasal cells. D492 generate luminal and myoepithelial cells in culture and in 3D rBM it forms elaborate branching structures reminiscent of TDLU in situ [23]. Interestingly, in this co-culture model we also observe spindle-like colonies indicating that BRENCs can induce EMT in D492 cells [46]. Isolation of these spindle like cells from D492 resulted in mesenchymal-like subline referred to as D492M (Fig. 3). D492M, shows a complete mesenchymal phenotype lacking epithelial markers such E-cadherin and cytokeratins but expressing mesenchymal markers such N-cadherin, vimentin, fibronectin, and FOXC2 [46].

## Cellular and Molecular Alteration During EMT

EMT has in recent years caught the attention of the clinical research community as an intrinsic mechanism in pathogenic processes such as fibrosis and progression of many types of cancer including breast cancer [47–49]. EMT is a term used to describe morphological change of epithelial cells from regularly arranged cuboidal, columnar or squamous form to a fibroblast or stellate appearance accompanied by increased occurrence of cell protrusions. This transition involves reorganization of the rigidly layered epithelium to a more loosely connected assembly of cells with increased migratory potential. Concurrently, each cell undergoing EMT rearranges its cytoskeleton allowing transformation to the mesenchymal trait resulting in a spindle-like phenotype. In developmental biology, EMT is a well-known theme, especially during gastrulation. Prior to gastrulation, the cell layer at the site of ingression has a typical epithelial arrangement characterized by apico-basal polarity, adherens junction and connection to basement membrane. The ingression is initiated by localized disruption of cell-to-cell adhesion altering cell morphology and causing breakdown of the basal membrane and finally, mass migration of the resulting mesenchymal cells into new territories. The sequence can be reversed as the migrating cells form settlements and new epithelial like layers form in a process often referred to as mesenchymal-to-epithelial transition (MET). Thus, EMT and MET represent recurrent programs enhancing cell and tissue plasticity necessary for the complex tissue remodeling during embryonic development [10].

One of the critical epithelial characteristics lost during EMT is cell-to-cell adhesion. This property is mediated by adherens junctions that are based on transmembrane proteins including cadherins, connecting adjacent cells via homophilic attachments made by their extracellular domains. Importantly, monitoring a switch from the expression of epithelial cadherin (E-cadherin) to a type of cadherin expressed by neurons and fibroblasts (N-cadherin), is the most commonly used method to assess EMT on a molecular level [50]. Alteration of cell-to-cell contact is also enforced through changes in desmosome expression allowing increased cell mobility. An important feature of EMT is the down-regulation of tight junction proteins including occludin and claudins constituting the trans-epithelial barrier [51]. In the normal tissue, occludin and claudins facilitate strong epithelial binding, and during EMT these proteins are progressively lost. In the breast epithelium, EMT is accompanied by a number of additional cytoskeletal changes including down-regulation of epithelial cytokeratins (CK5/6, CK8, CK14, CK17, CK18 and CK19), upregulation of vimentin, and smooth muscle actin, resulting in cells closely resembling myofibroblasts, a population of fibroblasts important for stromal tissue remodeling that are commonly activated during wound healing [52]. Finally, the EMT process is accompanied by loss of apical-basal polarity and anchoring junctions attaching the cells to the extracellular matrix and/or basement membrane. Cells that have undergone full EMT are practically indistinguishable from stromal fibroblasts [51].

Extracellular signals triggering EMT may channel through multiple pathways including the transforming growth factor beta (TGFβ), Notch, WNT, hedgehog, receptor tyrosine kinases (RTKs) and nuclear factor kappa-light chain enhancer of activated B-Cells (NFkB) [12, 53]. The most potent extracellular EMT inducer in breast epithelium and breast cancer is TGFß which promotes heterodimerization of TGFß receptors (Type I and II) following binding to the cell surface. TGFß ligand binding further triggers trans-phosphorylation of the cytosolic receptor domain which then enables them to phosphorylate downstream targets, the SMAD proteins. Phosphorylated SMADs dimerize in the cytosol, allowing entry to the nucleus and binding to promoter regions of target genes.

In view of the importance of EMT in promoting pathogenesis, identifying cells that may serve as paracrine source of TGFß or other EMT inducing ligands in cancerous or fibrotic tissue is being increasingly emphasized. Likely candidates include leukocytes, platelets, endothelial cells and cancer associated fibroblasts (CAFs) [54–57]. Immunohistochemical staining in breast cancer suggests that the microvessels in the vicinity of invading cancer cells may be a critical factor for adapting an EMT phenotype although the paracrine mechanisms contributing to this effect have not been fully elucidated [46].

The ability of TGFß to confer EMT seems to be context specific and in some models epithelial cells are susceptible to other factors. For instance, contact dependent activation of the Notch pathway through the ligand Jagged 2 has been shown to induce EMT in colorectal cancer cells that do not adapt the phenotype in response to TGFß treatment [58, 59]. The importance of Notch signaling in breast cancer is supported by studies showing up-regulation of Notch genes in metastases of the brain and clinical studies showing increased mortality in patients with high level co-expression of Notch and JAG1 in the tumors [59–61].

The canonical Wnt signaling pathway is directly related to the regulation of E-cadherin adhesion properties through control of its stabilizing partner ß-catenin. ß-catenin also functions as a transcription factor that can quickly transfer from cytosol to the nucleus in response to WNT signals where it activates promoters of target genes by displacing the co-repressor Groucho [62]. Wnt signaling molecules bind to the Frizzled family of receptors that associates with various co-receptors depending on ligand and cell type. The signal is further channeled to the cytosol through the canonical pathway affecting proteolytic control of ß-catenin or the non-canonical pathway that is independent of ß-catenin. These different pathways ultimately control transcriptional regulatory factors such as beta-catenin, SNAIL, SLUG, TWIST, ZEB1 and ZEB2 leading to increased expression of mesenchymal and decreased expression of epithelial markers [51].

MicroRNAs (miRNAs) have been implicated at several levels to be involved in the regulation of EMT [63]. Although a number of miRNAs have been shown to participate in EMT the miRNA-200 family is probably the best known [64]. The miR-200 family is an important regulator of epithelial integrity and loss of its expression is strongly associated with EMT. MiR-200 is a repressor of ZEB1 and ZEB2 that are the main EMT transcription factors that down regulate E-cadherin [65]. Recently, we demonstrated that miR-200c-141 induced MET when overexpressed in D492M. Furthermore, we also demonstrated that ectopic expression of miR-200c-141 in D492 and D492M prevented endothelial induced EMT. Thus, the miR-200 family and miR-200c-141 in particular are important caretakers of epithelial integrity in the human breast gland [66].

In a recent study we demonstrated that Sprouty-2 a conserved negative feedback regulator of receptor tyrosine kinases affected branching morphogenesis and EMT of D492 cells in 3D culture. In this study we showed that knocking down Sprouty-2 in D492 resulted in large hyperplasia-like branching structure and in coculture with endothelial cells almost all colonies displayed mesenchymal phenotype [67]. These data implicate Sprouty-2 as an important regulator in linking branching morphogenesis and EMT together and underlines the importance of 3D cultures in experiments when dealing with spatial and temporal biological events.

## Therapeutic Intervention of EMT and Cancer Stem Cell Phenotype

**Fig. 4:**
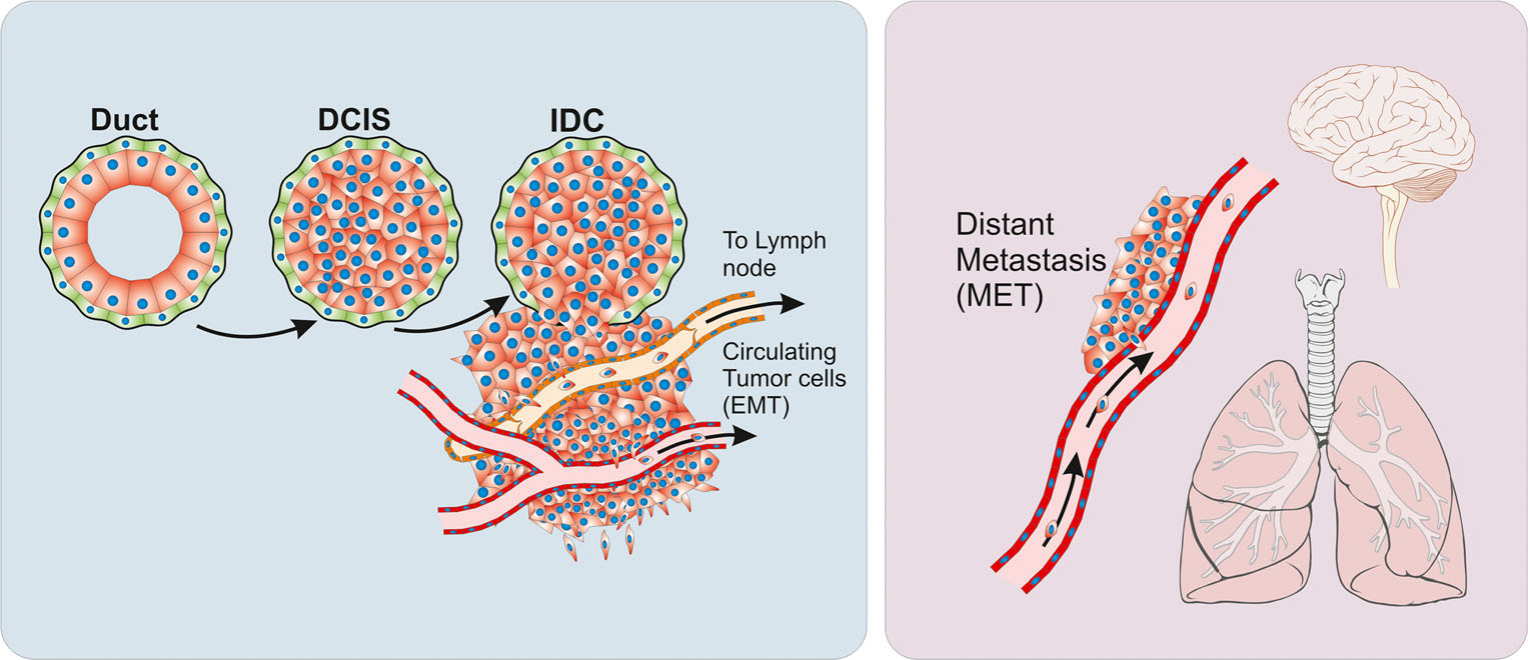
Cancer progression and metastasis. Breast cancer arises as localized lesions, most often within ducts and termed ductal carcinoma in situ (DCIS). These tumors are encapsulated by the myoepithelial cells (green) and a basement membrane (black line). When tumors progress to invasive disease the myoepithelial cells are progressively lost and the tumor breaks into the surrounding stroma, coming in contact with stromal cellular components, such as fibroblasts and microvessels. Changes to the microenvironment can induce cancer cells to undergo an epithelial to mesenchymal transition (EMT) a process where cells acquire increased survival and mobility. Increased mobility can lead to cells entering lymphoid or blood vessels and forming metastases in lymph nodes or distant organs. After forming metastases, established tumors often re-acquire epithelial characteristics through mesenchymal to epithelial transition (MET). “Lungs Diagram Simple” and “Brain Human Lateral View” by Patrick J. Lynch, licensed under Creative Commons Attribution 2.5

In epithelial cancer, the EMT phenotype is associated with increased aggressiveness and metastatic behavior [12] (Fig. 4). In breast cancer, studies have shown that EMT conversion occur preferentially in triple negative breast cancer including the basal-like subtype of breast carcinoma [48, 67, 68]. This subtype is characterized by a marker profile resembling a breast epithelial stem cell signature [69]. Al Hadjj et al. isolated a subpopulation of cells from breast cancer samples (CD44^high^/CD24^low^) and demonstrated by clonal inoculation into NOD/SCID mice that these cells contained cancer stem cells (CSCs) as observed by clonal growth and ability to regenerate the phenotype of the original tumor [70]. Mani et al. showed that in immortalized human breast epithelial cells (HMLE cell line), induction of EMT was associated with acquisition of stem cell properties as measured by increased ratio of CD44^high^/CD24^low^ expression [49]. Also, these cells have the ability to form mammosphere colonies in culture, a typical feature of breast epithelial stem cells [49]. It is, however, debated if the EMT phenotype is necessary for the occurrence of the cancer stem cell phenotype. Leth-Larsen et al. demonstrated that CD44^high^/CD24^low^ cell population with “epitheloid” phenotype rather than the CD44^high^/CD24^negative^ with mesenchymal phenotype is more efficient in generating tumors in nude mice [71]. It is, however not known if this particular cell population is prone to EMT. The same authors have also demonstrated the presence of tumor initiating cells within luminal epithelial tumors [72]. The relationship between epithelial and mesenchymal phenotype with regard to the cell-of-origin in breast cancer and metastatic potential is still unclear.

Typically, tissue stem cells proliferate slowly and are therefore more resistant to chemotherapy as many cancer drugs specifically target proliferating cells by inducing DNA damage, causing growth arrest and apoptosis. Survival statistics underlining increased mortality in breast cancer patients with mesenchymal elements in the primary tumor, e.g. triple negative and claudin-low breast cancers, suggests that EMT cells are less sensitive to chemotherapeutic agents. This is further supported by the finding that cell populations displaying an EMT and CSC phenotype are likely to be present within residual breast cancer after conventional chemotherapy [73].

At present there is not a full understanding of the inherent drug resistance of the EMT phenotype but several mechanisms could be at play. Firstly, the mesenchymal phenotype may be associated with higher expression of drug transporters such as ABCB1 (also known as multidrug resistance protein 1, MDR1) and ABCG2 that actively export cytotoxic drugs. Up-regulation of these protein pumps has been suggested as an important protection mechanism of the cancer stem cells that contribute to treatment failures and relapse. Although the EMT phenotype may be a step closer to stem cell character than the epithelial state, evidence supporting this mechanism is lacking and treatment of patients with ABCB1 inhibitors such as tariquidar and zosuquidar have not improved therapeutic response [74]. Another mechanism of how EMT may contribute to drug resistance includes altered cell cycle dynamics. Cells undergoing EMT may proliferate at lower rates or increase the proportion of quiescent cells, thereby minimizing the damaging effect of drugs targeting DNA. Moreover, EMT may favor migration of the cancer cells to a microenvironment that is not easily targeted by drugs. The blood–brain barrier is a simple example of such a restriction as it inhibits diffusion of macromolecules including the antibody based drug Herceptin. HER2 overexpressing cancer cells transpassing the blood–brain barrier prior to drug administration, possibly as consequence of EMT reprogramming, are thereby left untouched by the drug and may proceed to form metastatic colonies [75].

In addition to all the above mentioned mechanisms the number of gene expression changes in cells undergoing EMT may also directly involve genes affecting the sensitivity to apoptosis signals. In fact, accumulating data implicates key EMT transcription factors directly in acquisition of chemo- and radio-resistant cancer phenotype. As an example, a study of ovarian cancer cells suggest that key EMT regulatory factors Slug and Snail directly promote both chemo- and radio-resistance by suppressing p53-mediated apoptosis [76]. Snail has also been identified as a critical regulator of EMT in cisplatin-resistant lung cancer cells [77]. In breast cancer, another master EMT regulator, ZEB1, confers resistance to radiation therapy by allowing homologous recombination-dependent DNA repair through stabilization of CHK1. Interestingly, ZEB1 was found to be phosphorylated by ATM which is a key signaling protein in the DNA damage response pathway [78]. ZEB1 has additionally been identified as a mediator of chemo-resistance in models of lung and brain cancer and may therefore be considered a promising therapeutic target [79, 80]. Interestingly, ZEB1 has higher expression in basal-like breast cancer than luminal breast cancer. Basal-like breast cancers seem to have a higher proportion of CSC and they also have increased tendency to undergo EMT [81].

Since the TGFβ signaling pathway has been extensively linked to carcinogenesis and cancer progression, the pharmaceutical industry has been interested in developing drugs antagonizing its effect [82]. The relatively recent discoveries placing TGFβ at the centre of EMT and formation of metastatic disease further stimulates interest in targeting TGFβ pathways. Some of the recently discovered inhibitors are potentially useful in treating breast cancer e.g. an inhibitor of TGFβ type I and II receptors termed LY2109761 has been shown to reduce the metastatic formation of basal cell-like MDA-MB-231 breast cancer cells [83]. Another example is the recently discovered inhibitor EW-7195 that selectively targets the TGFβ type I receptor ALK5. This inhibitor dramatically reduces levels of phoshorylated Smad-2, expression of EMT phenotype and formation of lung metastases in xenograft mouse models following TGFβ stimulation [84].

Since EMT in normal and cancerous tissue is likely to be dynamically regulated by the epigenetic machinery, therapeutically targeting mechanism involved in regulating the epigenetic machinery is an attractive strategy. It has been shown that drugs (e.g. 5-azacytidine) that suppress normal promoter methylation inhibit EMT. The histone deacetylase inhibitor Panobinostat (LBH589) is being considered for the treatment of triple negative breast cancer and preliminary studies show that the drug prevents EMT in cell lines through its influence on ZEB transcription factors and E-Cadherin. Furthermore, this novel drug inhibits formation of metastasis by the breast cancer cell line MDA-MB-231 [85, 86]. The general effect of histone deacetylation antagonizes EMT by relieving suppression of important promoters including CDH1 (E-cadherin).

The drug Eribulin mesilate that influences microtubule assembly has been shown to improve survival in late-stage breast cancer patients through an unknown mechanism. A recent study suggests that the influence of the drug is mainly through suppression of mesenchymal conversion, further stressing the importance of cytoskeleton reorganization for the completion of EMT [87].

The anti-diabetic drug metformin, that is known to possess anti-cancer properties is reported to influence EMT. According to a recent study, this effect is probably due to activation of the AMPK pathway that is central to the regulation of energy metabolism and can be triggered by TGFβ signals [88]. A selective inhibitor of AMPK-alpha termed Compound C, abolishes the ability of TGFβ1 to induce apoptosis and EMT [89]. Recently, Hollier et al. demonstrated that FOXC2 transcription factor was efficient in inducing CSC and EMT phenotype in breast cancer cell lines. Interestingly, FOXC2 is expressed at high level in the claudin low breast cancer subtype associated with CSC and EMT phenotype [90]. Furthermore, they show that PDGFR-β is upregulated in FOXC2 induced EMT and that the FDA approved inhibitor of PDFGR, sunitinib reduced growth of CSC and metastasis.

## Closing Remarks

The branching epithelium of the adult female mammary gland undergoes repeated rounds of proliferation, differentiation and apoptosis during each menstruation cycle that is further exaggerated during pregnancy, lactation and involution. This continuous cellular remodeling has highlighted the importance of stem and progenitor cells within the mammary epithelium that provide the glandular epithelium with new functional cells. Furthermore, there is increasing interest in these stem cells, as they are believed to be targets for carcinogenic agents due to their longevity in tissues. It is debatable, however, whether or not it is the stem cells themselves, their progenitors or the more differentiated cells that have acquired stem cell properties and represent the true tumor initiating cells. It is of great importance that we are able learn more about the cellular hierarchy in the mammary gland and how the cross talk between the epithelium and stroma is maintained and how this goes awry during breast cancer progression. The inherent plasticity of the epithelium reflected in its ability to respond to external cues and change phenotype towards mesenchymal morphology via EMT is important, particularly since accumulating evidences implicates EMT in drug resistance and tumor relapse.

## Abbreviations

BRENCs: Breast endothelial cells
EMT: Epithelial to mesenchymal transition
ERα: Estrogen receptor α
FGF: Fibroblast growth factor
HUVEC: Umbilical vein endothelial cells
MET: Mesenchymal to epithelial transtion
miRNAs: MicroRNAs
MMPs: Matrix-metalloproteases
rBM: Reconstituted basement membrane
TDLU: Terminal duct lobular unit
TEB: Terminal end bud
VEGF: Vascular endothelial growth factor
